# Gaps in Hypertension Management in a Middle-Income Community of Quito-Ecuador: A Population-Based Study

**DOI:** 10.3390/ijerph19105832

**Published:** 2022-05-11

**Authors:** Patricia Ortiz, Yajaira Vásquez, Esperanza Arévalo, Patrick Van der Stuyft, Esteban Londoño Agudelo

**Affiliations:** 1 Facultad de Medicina, Pontificia Universidad Católica del Ecuador, Quito 170143, Ecuador; yajaira.vasquez@gmail.com (Y.V.); darevalo@puce.edu.ec (E.A.); 2 Consortium Latin-American Network for Multidisciplinary Research on Chronic Non-Communicable Diseases, Medellin 050010, Colombia; patrick.vanderstuyft@ugent.be (P.V.d.S.); elondono@ces.edu.co (E.L.A.); 3 Departamento de Pediatría, Obstetricia y Ginecología y de Medicina Preventiva, Universidad Autónoma de Barcelona, 08193 Barcelona, Spain; 4 Department of Public Health and Primary Care, Faculty of Medicine and Health Sciences, Ghent University, 9000 Ghent, Belgium; 5 Department of Public Health, Institute of Tropical Medicine, 2000 Antwerp, Belgium; 6 Grupo de Epidemiología, Facultad Nacional de Salud Pública, Universidad de Antioquia, Medellín 50010, Colombia; 7 Facultad de Medicina, Universidad CES, Medellín 0510, Colombia

**Keywords:** hypertension, primary health care, hypertension management, health systems, Ecuador

## Abstract

Optimal hypertension care and control at population level significantly reduces cardiovascular morbidity and mortality. The study objective was to measure the gaps in the diagnosis, care, and control of hypertension in residents of an urban community in Quito, Ecuador. A cross-sectional population-based study with a sample of 2160 persons was performed using a survey and direct blood pressure measurement. Logistical regression models were used for analyzing factors associated with the gaps, expressed as percentages. The prevalence of hypertension was 17.6% [CI 95% 17.3–17.9%]. The diagnosis gap was 6.1% [CI 95% 5.9–6.2%] among the entire population and 34.5% [CI 95% 33.7–35.3%] among persons with hypertension. No access gaps were detected; whereas the follow-up gap was 22.7% [CI 95% 21.8–23.6%] and control gap reached 43.5% [CI 95% 42.6–44.2%]. Results indicated that being male, older than 64 years, an employee, without health insurance, and not perceiving a need for healthcare, increased the risk of experiencing these gaps. Data showed appropriate access to health services and high coverage in the diagnosis was due to the application of a community and family healthcare model. Notwithstanding, we found significant gaps in the follow-up and control of hypertensive patients, especially among older males, which should warrant the attention of the Ministry of Health.

## 1. Introduction

Hypertension (HTN) is the most important risk factor for disease burden and premature death worldwide [1,2]. It affects approximately one billion people, three-fourths of whom live in low- and middle-income countries (LMIC) [3], and it causes more than ten million deaths per year, most of which are premature and preventable [4].

HTN affects 20–40% of adults in the Americas Region and represents 6.6% of the disability-adjusted life- years (DALYs) [5,6]. Fifty-nine percent of deaths due to ischemic heart disease and 63.2% of cerebrovascular events in Latin America are attributed to HTN in people between 50 and 69 years of age [1,7]. In Ecuador, ischemic heart disease was the main overall cause of death in 2018, accounting for 11.1% of all deaths in both sexes. Hypertensive diseases, ischemic heart and cerebrovascular diseases caused 15,619 deaths, which represents 22% of total deaths in the country [8]. The death rate from cardiovascular diseases (CVDs) in Ecuador was 131.6 per 100,000 people in 2016, from which 39.7% were deaths occurred under 70 years of age [9].

Efficient and equitable health systems are required to decrease the HTN-related disease burden. Globally, health systems should guarantee universal coverage, promote primary prevention, and provide quality secondary prevention, to assure HTN treatment and control [10,11]. Evidence shows that HTN control may significantly contribute to decreasing morbidity and mortality due to CVDs. Despite this, the global burden of HTN is currently increasing [12,13,14].

A significant proportion of people with HTN is not aware of their condition; among those who are aware, only a minority have been treated and controlled [15,16]. It is estimated that approximately 80% of people with HTN in the Americas and the Caribbean region do not have adequate control of their blood pressure (BP) [5,17]. Another combined international study on HTN care in Argentina, Brazil, Chile, and Colombia found that only 57% of hypertensive people were aware of their condition. Of these, 53% received treatment, and only 30% had their BP under control [18].

According to data from the STEPS survey (2018) [19], the prevalence of HTN in Ecuador was 20% nationwide. A total of 45.2% of people aged 18 to 69 years with HTN were not aware of their condition, and 12.6% were aware of their diagnosis but did not take antihypertensive medication.

In Ecuador, chronic patient management is guided by the Integral Health Care Model (IHCM), which, based on the Primary Health Care strategy, strengthens the resolutive capacity of the first- and second-line health care services. This is complemented with the “Physicians in the Neighborhood” strategy, which is geared towards guaranteeing integral health care for priority groups, such as those with chronic non-communicable diseases [20]. There are Clinical Practice guidelines and protocols for professionals at the three levels of healthcare for the management of HTN [21].

Using a populational perspective, this study measures the existence, magnitude, and main determinants for the access, diagnosis, treatment, follow-up and control gaps affecting the health care of persons aged 35 to 70 years with HTN residing in an urban middle-income community in Quito, Ecuador.

## 2. Materials and Methods

### 2.1. Study Design

A population-based, cross-sectional study, including adult population between 35 to 70 years of age, living in the urban area of the Conocoto community in the Metropolitan District of Quito, between July 2015 and June 2016.

### 2.2. Study Site and Population

The Ecuadorian Health System is composed of a public and a private sector. The public sector provides health care to the non-insured population. It includes the Ministry of Public Health (MSP), the Ministry of Economic and Social Inclusion (MIES) and the municipalities. The social security institutions [Ecuadorian Institute of Social Security (IESS), Armed Forces Social Security (ISSFA) and the National Police Social Security (ISSPOL)] provide health care to affiliated workers and their families. In 2018, the population covered by the IESS was 27.3%, the special insurance schemes for the army and police force (ISSFA and ISSPOL) reached 1.7%, and private insurance companies covered 9%. This left more than 60% of the population not formally covered by any insurance scheme. This population is entitled to receive free healthcare services run by the Ministry of Health [22]. These theoretical figures contrast with the strong private offering, which allows patients to turn to private services (including pharmacies) whenever they want and at any level of care, so long as they can fully pay for it, regardless of the coverage scheme they belong to [23]. Information disaggregated at community-level could not be found on this issue.

Conocoto is a middle-income community that forms part of the Metropolitan District of Quito. It is located 6.8 miles from downtown, with an area of 51.46 km^2^. According to the 2010 census [24], the total population was 82,072 inhabitants (52% women), with an average age of 29 for men and 31 for women. People older than 70 years account for 4% of the population and 82.7% of the population is within the category of working age. According to poverty indicators, there is a high percentage of people classified as above the poverty line (72%); 87% of the population consider themselves “mestizo”, while 4.98% self-identify as Afro Ecuadorians, indigenous or mulatto [25].

The selection criteria for the study site were urban setting, low- or middle-income population, health service functionally similar to that of health establishments nationwide, and a commitment by local health authorities to develop an intervention based on the study results.

### 2.3. Sample Size and Sampling Strategy

The sampling frame for this study was derived from the VII Population Census and the VI Housing 2010 of Ecuador, and the updated mapping elaborated by the National Census and Statistics Institute (INEC), which contained a complete listing of occupied housing and population for the Conocoto community. The sample design was probabilistic by conglomerates, defined as the registered sectors (N = 172) and proportional to their population size. A two-stage sampling method was used. The primary sampling unit (PSU) was the census sectors (10 randomly selected). The secondary selection unit was an inhabited dwelling with people between 35 and 70 years of age, and the observation unit was the household (group of people who live together and share a “common pot” of meals and where biological reproduction and social processes take place; housing refers to the physical structure, where more than two households may live [26].

The PSU and housing were selected by simple random sampling, taking into account an average of 1.75 persons between 35 and 70 years of age in each household. A total of 2237 housings were selected from a sampling frame of 16,030 and four housing units per PSU as a replacement. Finally, all household members between the ages of 35 and 70 were surveyed.

The sample size was calculated with an expected prevalence of hypertension of 25% among the population between 35 and 70 years of age, with a confidence level of 95%, a precision of 5%, design effect of 1.5, and a non-response rate of 4%, rendering a total of 2100 persons.

### 2.4. Study and Measurement Procedures

Two trained surveying teams made door-to-door visits to all selected households and dwellings. The eligible subjects—individuals between 35 and 70 years of age—who gave their informed consent to participate in the study were interviewed using a previously validated structured questionnaire. If the person was not at home at the time of the interview, two further attempts were made to locate a person who met the inclusion criteria for age.

Information was gathered by means of a “Survey form for the study of gaps in Hypertensive Care Management”. The survey included several sections covering general household and subject data, namely: sociodemographic data, use of health services, lifestyle: tobacco, alcohol consumption and physical activity, HTN diagnosis and treatment, and BP figures.

BP was measured using a digital device. Following the Panamerican Health Organization (PAHO) recommendations for population-based studies [27], three BP measurements were carried out. The first reading was discarded, and an average was obtained between the second and third readings.

A hypertensive person with a previous diagnosis was defined as a person diagnosed as hypertensive by a physician, regardless of the BP values at the time of the survey.

A presumptive hypertensive person was defined as a person without a previous diagnosis of HTN, who at the time of the survey had systolic tension values of ≥140 or diastolic tension values of ≥90.

Persons with no evidence of HTN were defined as those who referred not having a previous diagnosis of HTN and who had BP values of <140/90 [28].

Box 1 shows the definitions used in the study as the basis to calculate gaps.

Box 1 **Definitions of Needs and Gaps**

Need for medical care: proportion of people who had an illness, health discomfort or accident, and who required medical attention.

Search for medical care: proportion of people who took action to solve their health problem.

Access Gap (AG): proportion of people who reached a health service provider and were not attended to in each subpopulation (persons with a previous hypertension diagnosis, presumptive hypertensive patients, and population with no evidence of hypertension).

Diagnosis Gap (DG): proportion of people who did not report a previous hypertension diagnosis, and who presented average BP values ≥140 systolic or ≥90 mmHg after the two BP measurements at the time of examination, or when the survey was applied, among the total of surveyed people.

Follow-up Gap (FG): proportion of people with a previous HTN diagnosis who reported that during the last year they have not attended health services for their HTN.

Treatment Gap (TG): proportion of people with a previous HTN diagnosis who reported having received a prescription for anti-hypertensive medication but did not get or take the treatment.

Population Control Gap (PCG): proportion of people ≤59 years with or without a previous hypertension diagnosis, who at the time of the survey presented BP values ≥140/90; or people ≥60 years with BP values ≥150/90; as well as people with a previous diabetes diagnosis, with BP ≥140/90 (regardless of age).

Hypertension Control Gap (HCG): proportion of people ≤59 years with a previous HTN diagnosis, who at the time of the survey presented BP values ≥140/90, or people ≥60 years with BP values ≥150/90; as well as people with a previous diabetes diagnosis with arterial pressure ≥140/90 (regardless of age).

### 2.5. Statistical Methods

Data processing and analysis were carried out using the SPSS v24 software. Once raw data was entered into the program, the data entry assistants validated the incorrect and missing values, enumerated in an “observations log” to be delivered to the supervisors. Secondly, incorrect data and erroneously recorded and missing information were corrected using the validation grids established in SPSS. For the data entry validation, a random sample of the surveys entered was verified and contrasted with the forms. This resulted in a 2% error in data entry.

Previous to the data processing and analysis, expansion factors were calculated to extrapolate sample data for the population (i.e., to expand the sample) [29]. These factors were calculated according to selection probability determined by the sampling design for each sample unit. The expansion factor was defined as the inverse of the probability of choosing a household and its members in a determined study domain [30]. Incidents registered during data gathering, such as lack of reply, rejection, no one home, were considered to adjust the expansion factors according to survey coverage.

In both the descriptive and analytical phases, data analysis was carried out based on the population values. Gaps and socioeconomic characteristics were calculated, expressed in absolute frequencies and total percentages; continuous variables were expressed as means and standard deviation.

## 3. Results

A total of 1583 surveys were performed in households from the Conocoto community. A total of 2160 people between the ages of 35 and 70 were found, which represented 78,591 individuals in the expanded population. Table 1 displays the sample and expanded population features within each sub-population: hypertensive with a previous diagnosis, presumptive hypertensive, and without evidence of HTN.

There were more females in all sub-populations, except for the presumptive hypertensive group. The average age was higher in the sub-population with a previous diagnosis of HTN (59.07 ± 7.32 years) when compared to the average age for the presumptive hypertensive sub-population (55.8 ± 9.7 years) and the sub-population without evidence of HTN (50.2 ± 9.9 years). The predominant self-described ethnic group was *mestizo* in all sub-populations. The proportion of unemployed people was higher in the group of those previously diagnosed as hypertensive. The proportion of those not affiliated with a health insurance system was higher in the known and presumptive hypertensive groups (Table 1).

### 3.1. Gaps in the Process of Hypertension Care Management

An overall hypertension prevalence of 17.6% [CI 95% 17.3–17.9] was found.

Gaps in access to healthcare were not observed in any of the sub-populations. The lack of perceived need, as a requirement for healthcare demand, was lower in the sub-population of those previously diagnosed as hypertensive (6.2% CI 95% 5.74–6.7), as compared to the sub-populations of those presumptive hypertensive and without evidence of HTN (33.2% and 31.8%, respectively).

The diagnosis gap was 6.1% [CI 95% 5.9–6.2] among the population surveyed and 34.5% [CI 95% 33.7–35.3] among the hypertensive population. The pharmacological treatment gap registered a proportion of 1.7% [CI 95% 1.37–1.95], while the gap in adherence—self-referenced, after the Morinsky test [31] was 59.5%. The follow-up gap obtained was 22.7% [CI 95% 21.8–23.6]; the control gap in the population with a previous HTN diagnosis was 13.7%. Meanwhile, the control gap on a population level was 43.5% [CI 95% 42.6–44.2].

### 3.2. Factors Associated with Gaps in Diagnosis, Follow-Up, and Control

Associated factors were analyzed separately for each gap. For the diagnosis gap, it was observed that males, those living alone, those with fewer years of schooling, and employees presented a greater gap. On the contrary, people under 65, the population self-identified as Afro-Ecuadorian, indigenous, or mulatto, and people with diabetes diagnosis showed a lower gap (Table 2). Not having health insurance seems to be a protective factor for the diagnosis gap, even though its OR value is close to 1. Table 3 shows associated factors related to the follow-up gap, revealing that people under 65 with no health insurance affiliation and with diabetes had a larger gap. On the other hand, males, people living alone, and those employed presented a lower gap. There was no indication that Afro-Ecuadorian, indigenous, or mulatto had a greater follow-up gap, probably because only a minimal proportion of the population of Conocoto self-identified with these ethnic groups.

Concerning the factors associated with the population’s control gap, it was observed that males, people living alone, those employed, and those not affiliated with health insurance services presented a higher control gap. In contrast, people under 65 old years, with diabetes, and who do not exercise had a lower gap. No associations were found with tobacco and alcohol consumption or with ethnicity (Table 4).

The logistic regression model (Table 5) indicates the predictive associated factors for the different gaps, like those observed in the bi-varied analysis. For the diagnosis gap, the main associated factors were men living alone, without health insurance affiliation, employed, and who avoided seeking medical attention. Being under 65 years of age constituted a protective condition. For the follow-up gap, the associated factors were being under 65, without health insurance affiliation, and with a diagnosis of diabetes. On the other hand, being a working male constituted a protective factor. Factors associated with the control gap were being male, without health insurance affiliation, employed, those who did not feel the need to seek out health care attention, and those who did not exercise. Together, these factors explain 62% of the control gap. People having a diagnosis of diabetes and adequate follow-up care had a smaller probability of uncontrolled HTN.

## 4. Discussion

This study revealed an HTN prevalence of 17.6%, the non-existence of access gaps to healthcare among the Conocoto community residents, and a relatively small diagnosis gap (6.1%). On the other hand, the follow-up gap (27.7%) and the control gap at a populational level (43.5%) should both warrant the attention of the Ministry of Health.

This HTN prevalence value is similar to that reported in the STEPS survey performed in 2018 [19], with a nationwide prevalence of 20%. However, this data differs from results reported in different country areas, which showed large differences in the HTN prevalence [32,33,34,35,36]. This variability can be explained by the various definitions and measurement methods used [2,37,38]. Our results suggest that the diagnosis of HTN in Ecuador has improved over the last decade. Nevertheless, the prevalence of HTN among the researched population is relatively low compared to the average reported for Latin American countries [39].

The inexistence of access gaps could at least partially result from the implementation of integral health reforms undertaken by Ecuador during the past decade and the existence of a public policy aimed at regulating health care [40]. The 2008 Constitution [41] recognizes health as a human right and the State as a guarantor of this right. Since then, a series of socially focused policies, plans and programs have been formulated, directed toward providing ongoing and timely universal access to integrated health care. To comply with the Organic Health Law (2006) [42], the Ministry of Public Health (MSP) mandates free healthcare at all levels of the public healthcare network [41]. Granda and Jimenez [43] found that between 2006 and 2014, there was a decrease in inequity in access to healthcare. This could be attributed to the reform of the Ecuadorian health system, evidenced by broader use of public healthcare service’s curative care by the first and third socioeconomic quintile of the population. Nevertheless, obstacles that cause insufficient care in health services persist, preventing the delivery of consistent, adequate and high-quality care to achieve long-term control of chronic diseases [44].

The diagnosis gap in the population was significantly lower (6.1%) than those reported in other countries, such as India [45], Korea [46], The United States, Canada, and England [18]. Even in high-income countries, 37% of hypertensive patients do not acknowledge their disease [47]. The notable results regarding diagnosis coverage may be rooted in the previously mentioned health system reforms, but they should be explored in future studies. Findings on the sociodemographic factors associated with this gap are consistent with those of other studies [45]: males face multiple barriers in accessing healthcare, which, added to the lack of routine BP control and few support networks, prevent proper diagnosis. The traits traditionally considered inherent to masculinity, such as self-sufficiency, self-dependence and/or strength, make men more prone to not seek healthcare, to seek it out following a delay, and to develop complications associated with these delays [48,49].

Although no specific explanations were found for the influence of living alone on the diagnosis gap, some studies show an association between the absence of support networks, whether friends or family and an increase in systolic BP [50]. On the contrary, support networks have been positively associated with treatment compliance and better self-care behavior in patients with HTN [51,52].

The follow-up gap in this study was 27.7%. It was larger for females under 65, with no social security affiliation, unemployed, and having a diabetes diagnosis. Follow-up constitutes an opportunity to make a clinical decision based on BP values, provide counseling, and evaluate treatment adherence [53]. The absence of follow-up may result in poor disease control, greater risks for hospitalization, decreased clinical efficiency and higher mortality [54,55]. Several barriers have been identified for optimal follow-up. However, the evidence contradicts [54]. In the first level of the Ecuadorian public health system, appointment scheduling does not allow for the health care professional to be selected. The resulting frequent change in physicians hampers care continuity, obstructing adequate follow-up for patients with chronic diseases.

Despite women’s unquestionable progress in participation in work, politics and education, inequity nonetheless persists; women, particularly those who are poor, have less access to health care resources within and outside of their families and less decision-making power over their own health. Furthermore, women frequently engage in informal employment and are responsible for their dependents’ care [56]. This limits their ability to access social security benefits, which may constitute a barrier to continuing with follow-up consultations.

The control gap in people with previously diagnosed HTN and the overall population (13.7% and 43.5%, respectively) showed significantly lower values than those reported in a study performed in 2017 in Cuenca, Ecuador. In the latter, 51.1% of the individuals with HTN diagnosis had adequate control [33]. Conversely, the STEPS (2018) [19] survey reported that 26% of those who knew their diagnosis and took medication had their blood pressure under control. Likewise, the PURE study showed that the rate of non-controlled hypertension in South America was 81.2% of the population previously diagnosed with HTN [18]. In a systematic review [15], this data corresponded with 66.8% for Central America, South America, and the Caribbean. North America had values of 49.9% for men and 44.1% for women. While HTN control has increased substantially since 2000 in high-income countries, from 18% to 28% in 2010, it remains insufficient [46].

The apparent contradiction between the results obtained regarding the gap of follow-up and the gap in population control may be explained due to the fact that the patients could have a scheduled appointment available for the follow-up (definition of follow-up gap), however the quality of the consultation is not always the best due to the lack of application of the GPC recommendations, such as appropriate counseling about habits and healthy styles, treatment standardization, adherence to treatment evaluation, as well as the lack of fulfillment of the recommendations on behalf of the patients, which finally affect arterial pressure control.

The diagnosis, follow-up, and control gaps of the population with HTN require primary and secondary prevention actions to avoid complications or major cardiovascular injuries and decrease quality of life [57]. In addition, strategies are required to improve healthcare-seeking behavior; although mainly driven by individual decision, this behavior is mediated by an effective response from the health services. Examples of the latter are the availability of medical appointments in a timely fashion and at a convenient time for patients who work, a sustained accompaniment on behalf of the health professionals to improve self-care, and support for identifying adequate solutions for the problems. A healthcare plan developed with the user, and the definition and monitoring of the fulfilment of goals could help in this endeavor [58].

Undisputedly, health care alone is not enough to formulate a complete approach on HTN since many of the implicated factors extend beyond health systems’ scope. By tackling some socio-economic determinants, they might better impact the population health [59,60]. To control HTN, it is necessary to account for an integrative response based on inter-sectoral collaboration and integrated methods of caring for persons with chronic conditions. Self-care strengthening, redefinition of roles and responsibilities of doctors, nurses, and social workers, and incorporating adequate financial models, represent fundamental elements of this integrative care model [47,61].

Methodological limitations in the current study are related to HTN measurements carried out on a single day, which might cast false positive or negative results. In order to reduce this possibility, three serial BP measurements were made, observing PAHO’S recommendations for population studies [27]. The selection criteria of including only participants aged between 35 and 70 years of age, excluded an important part of the population. People over 70 years most likely live in worse socio-economic and health conditions and might exhibit larger gaps regarding medical care and control of their hypertension. Nevertheless, this age group is relatively small and represents less than 4% of the total country’ population [25,29]. It is relevant to acknowledge that this study did not explore factors related to the frequency nor quality of follow-up. The values found in this study cannot be generalized to the entire Quito Metropolitan District population, let alone on a national level. It must be considered that Ecuador is a heterogeneous country, not only in its geographical, ethnic, and cultural diversity, but also due to the pronounced social and economic imbalance and the availability and organization of its health services.

## 5. Conclusions

This study demonstrated the existence of good access and coverage for diagnosis and treatment and problems with the follow-up and control of persons with HTN. This could be explained by the persistence of a healthcare model focused on acute diseases’ clinical management. The absence of a follow-up system that guarantees healthcare continuity in patients with chronic diseases, especially diabetes and hypertension, indicates this situation. A lack of follow-up is also associated with deficiencies in scheduling appointments and a healthcare service wherein organization of ongoing care is focused on freedom of choice. The gaps identified by this study expose the current limitations of the national healthcare system and its model.

## Figures and Tables

**Table 1:** Sociodemographic characteristics of the urban population of the community of Conocoto. 2016.

		Known Hypertensive			Presumptive Hypertensive			Population without Evidence of Hypertension			Total		
		9060	240	11.5%	4766	115	6.1%	64,765	1805	82.4%	78,591	2160	100%
		Population (N)	Sample (n)	% (N)	Population (N)	Sample (n)	% (N)	Population (N)	Sample (n)	% (N)	Population (N)	Sample (n)	% (N)
Gender	Male	2452	112	27.1%	3344	78	70.2%	26,275	855	40.6%	32,071	1045	40.8%
	Female	6608	128	72.9%	1422	37	29.8%	38,490	950	59.4%	46,520	1115	59.2%
Who they live with	Alone	842	16	9.3%	671	8	14.1%	3418	98	5.3%	4931	122	6.3%
	Accompanied	8218	224	90.7%	4095	107	85.9%	61,347	1707	94.7%	73,660	2038	93.7%
Ethnicity	Indigenous, Afro-Ecuadorian, Montubio	103	5	1.1%	29	1	0.6%	4176	62	6.45%	4308	68	5.48%
	Mestizo and Caucasian	8957	235	98.9%	4737	114	99.4%	60,589	1743	93.55%	74,283	2092	94.52%
Employment	Workers	4261	116	47.0%	3556	81	74.6%	40,997	1203	63.3%	48,814	1400	62.1%
	Non-worker	4799	124	53.0%	1210	34	25.4%	23,768	602	36.7%	29,777	760	37.9%
Health insurance affiliation	No	4656	106	51.4%	2772	57	58.2%	31,778	829	49.1%	39,206	992	49.9%
	Yes	4404	134	48.6%	1994	58	41.8%	32,978	976	50.9%	39,376	1168	50.1%
Age	Under 65 years	7087	173	78.2%	3440	97	72.2%	58,392	1655	90.2%	68,919	1925	87.7%
	Over 65 years	1973	67	21.8%	1326	18	27.8%	6374	150	9.8%	9672	235	12.3%
Means Age (SD)		59.07 ± 7.32			55.8 ± 9.7			50.2 ± 9.9			51.5 ± 10.1		
Years Approved (MeansSD)		11.0 ± 5.34			11.3 ± 4.6			12.1 ± 4.7			11.9 ± 4.8		
Means Systolic pressure (SD)		127.1 ± 16.4			142.9 ± 11.8			114.4 ± 10.3			117.6 ± 13.5		
Means Diastolic pressure (SD)		76.4 ± 10.5			88.1 ± 7.1			71.9 ± 8.3			73.4 ± 9.4		

**Table 2:** Associated Factors to the Diagnostic Gap.

Variable	Category	Population = 78,591						
		Population (N)	Gap (N)	%	OR	LB	UB	VALUE *p*
**DIAGNOSTIC GAP** **(People with undiagnosed HTN)**		78,591	4766	6.1		5.89	6.23	
Gender	Male	32,071	3344	10.4	3.692	3.464	3.935	<0.001
	Female	46,520	1422	3.1				
Age groups	Under 65 years old	68,919	3440	5.0	0.331	0.309	0.354	<0.001
	Over 65 years old	9672	1326	13.7				
Who they live with	Alone	4931	671	13.6	2.676	2.452	2.92	<0.001
	Accompanied	73,660	4095	5.6				
Ethnicity	Afro-Ecuadorians, Montubio, Indigenous	4308	29	0.7	0.1	0.069	0.144	<0.001
	Mestizo, Caucasians	74,283	4737	6.4				
Employment	Workers	4261	865	20.3	2.979	2.619	3.389	<0.001
	Non-workers	4799	378	7.9				
Health insurance affiliation	No	4656	580	12.5	0.804	0.713	0.9	<0.001
	Yes	4404	662	15.0				
Diabetes diagnosis	Yes	2179	243	11.2	0.738	0.636	0.857	<0.001
	No	6882	1000	14.5				
		**N**	**Means**	**Standard deviation**				**VALUE *p***
Education (years passed approved)	Yes Gap	1247	11.1	5.1				<0.001
	No Gap	7828	11.9	4.8				

Lower and upper bounds (LB, UB) of the 95% confidence interval.

**Table 3:** Follow-up gap, associated factors.

Variable	Category	Total Hypertensive Patients with Previous Dx (N = 9060)						
		Population (N)	Gap (N)	%	OR	LB	UB	VALUE *p*
Follow-up gap		9060	2057	22.7		21.8	23.6	
Gender	Male	2452	393	16.0	0.56	0.5	0.64	<0.001
	Female	6608	1665	25.2				
Age group	Under 65 years old	7087	1882	26.6	3.69	3.13	4.34	<0.001
	Over 65 years old	1973	176	8.9				
Who they live with	Alone	842	29	3.4	0.11	0.08	0.16	<0.001
	Accompanied	8218	2029	24.7				
Ethnicity	Afro-Ecuadorian, Montubio, Indigenous	103	0	0.0	N/A			N/A
	Mestizo, Caucasian	8957	2057	23.0				
Employment	Worker	4261	577	13.5	0.35	0.32	0.39	<0.001
	Non-worker	4799	1481	30.9				
Health insurance affiliation	No	4656	1413	30.3	2.53	2.28	2.8	<0.001
	Yes	4404	645	14.6				
Diabetes diagnosis	Yes	2178	1028	47.2	5.08	4.56	5.65	<0.001
	No	6881	1029	15.0				

Lower and upper bounds (LB, UB) of the 95% confidence interval.

**Table 4:** Associated factors to the Control Gap at the population level.

Variable	Category	Known Hypertensive + Presumptive Hypertensive						
		Population (N)	Gap (N)	%	OR	LB	UB	VALUE *p*
Control gap		13,826	6008	43.5		42.6	44.3	
Gender	Male	5796	4066	70.20	7.37	6.83	7.95	<0.001
	Female	8029	1942	24.20				
Age group	Under 65 years old	10,526	4379	41.60	0.73	0.68	0.79	<0.001
	Over 65 years old	3299	1629	49.40				
Who they live with	Alone	1513	751	49.60	1.32	1.19	1.47	<0.001
	Accompanied	12,312	5257	42.70				
Ethnicity	Afro-Ecuadorians, Montubio, Indigenous	132	58	43.90	1.02	0.72	1.44	0.91
	Mestizo, Caucasian	13,695	5951	43.50				
Employment	Workers	7817	4421	56.60	3.63	3.37	3.90	<0.001
	Non-workers	6008	1587	26.40				
Health insurance affiliation	No	7428	3352	45.10	1.16	1.08	1.24	<0.001
	Yes	6399	2657	41.50				
Diabetes diagnosis	yes	2824	888	31.40	0.53	0.48	0.58	<0.001
	No	11,002	5120	46.50				
Exercise	No	9066	3878	42.80	0.92	0.86	0.99	0.03
	Yes	4760	2130	44.70				
Cigarette Consumption	yes	6428	2490	38.70	1.01	0.91	1.12	0.85
	No	2047	788	38.50				
Alcohol Consumption	Yes	0.00	0.00	0.00				NA
	No	13,825	6008	43.50				

Lower and upper bounds (LB, UB) of the 95% confidence interval.

**Table 5:** Logistic regression model of risk factors for gaps in diagnosis, monitoring and control of hypertension.

Gaps	Risk Factors	OR Adjusted	Confidence Intervals		Significance
Diagnostic gap	Gender (male)	3.14	2.910	3.406	0.001
	Age < 65 years	0.26	0.239	0.283	0.001
	Live alone	2.49	2.229	2.786	0.001
	Affiliation (No)	2.61	2.418	2.821	0.001
	Employment (workers)	3.08	2.813	3.387	0.001
	Not Attention seeking	2.15	1.686	2.761	0.001
Follow-up gap	Gender(male)	0.7	.655	0.900	0.001
	Age < 65 years	3.95	3.246	4.814	0.001
	Affiliation (No)	2.19	1.936	2.496	0.001
	Employment (workers)	0.715	0.617	0.829	0.001
	Diabetes diagnosis (yes)	4.16	3.625	4.785	0.001
Control gap	Gender (male)	4.8	4.34	5.4	0.001
	Affiliation (No)	1.62	1.46	1.81	0.001
	Employment (workers)	1.63	1.45	1.84	0.001
	Need for attention (No)	6.68	5.55	8.05	0.296
	Diabetes diagnosis (yes)	0.39	0.32	0.42	0.001
	Exercise (No)	1.63	1.43	1.43	0.01
	Follow-up gap	0.15	0.12	0.19	0.001

## Data Availability

Data associated with this study are available upon request from the corresponding author.
